# Phage display peptide libraries: deviations from randomness and correctives

**DOI:** 10.1093/nar/gky077

**Published:** 2018-02-06

**Authors:** Arie Ryvkin, Haim Ashkenazy, Yael Weiss-Ottolenghi, Chen Piller, Tal Pupko, Jonathan M Gershoni

**Affiliations:** Department of Cell Research and Immunology, George S. Wise Faculty of Life Sciences, Tel Aviv University, Tel Aviv 69978, Israel

## Abstract

Peptide-expressing phage display libraries are widely used for the interrogation of antibodies. Affinity selected peptides are then analyzed to discover epitope mimetics, or are subjected to computational algorithms for epitope prediction. A critical assumption for these applications is the random representation of amino acids in the initial naïve peptide library. In a previous study, we implemented next generation sequencing to evaluate a naïve library and discovered severe deviations from randomness in UAG codon over-representation as well as in high G phosphoramidite abundance causing amino acid distribution biases. In this study, we demonstrate that the UAG over-representation can be attributed to the burden imposed on the phage upon the assembly of the recombinant Protein 8 subunits. This was corrected by constructing the libraries using *supE44*-containing bacteria which suppress the UAG driven abortive termination. We also demonstrate that the overabundance of G stems from variant synthesis-efficiency and can be corrected using compensating oligonucleotide-mixtures calibrated by mass spectroscopy. Construction of libraries implementing these correctives results in markedly improved libraries that display random distribution of amino acids, thus ensuring that enriched peptides obtained in biopanning represent a genuine selection event, a fundamental assumption for phage display applications.

## INTRODUCTION

Thirty years ago George Smith introduced ‘phage display’ as a means to express vast collections of recombinant proteins and peptides to be screened by affinity selection (1) (a process coined ‘biopanning’ (2)). Two basic systems evolved: (i) phage display of random peptide libraries and (ii) the production of phage displayed antibodies. The latter has been used extensively to generate human monoclonal antibodies (3–6), replacing to some extent the production of murine monoclonal antibodies via classical hybridoma technology. The first system, random peptide libraries, was initially applied to the epitope analysis of a specific monoclonal antibody with the ultimate intent of affinity selection of a peptide-mimetic assumed to simulate and represent the cognate epitope of the antibody being studied (7,8). Cortese *et al.* went on to implement random peptide phage display to study polyclonal sera, demonstrating that pathogen related peptides could be isolated through biopanning peptide-libraries with disease-defining polyclonal serum (9–14). In many cases the application of phage display libraries was performed to clone out a specific peptide or antibody for further development and study. More recently, phage display has also served as the basis for computational prediction of epitope-structures (15–21), as reviewed by Sun *et al.* (22) and for the critical profiling of the repertoire of antibody specificities of polyclonal sera, i.e. profiling the IgOme (23,24), see also (25).

An implicit assumption in phage displayed based applications is that the library to be screened truly represents an unbiased comprehensive collection of peptides from which affinity selection can proceed. The selected peptides can be taken, therefore, to faithfully represent the structural idiosyncrasies and/or specific binding activities of the antibody (or antibodies) being analyzed. Deviations from this inherent assumption can negatively affect analyses in several ways: (i) over-representation of a given peptide can increase its probability to be affinity selected or non-specifically enriched; (ii) severe under-representation of peptides obviously prevents the selection of otherwise meaningful peptides missing from the library; (iii) in computational applications when a collection of peptides serves as a database, the *a priori* assumption that the isolated peptides genuinely represent an objective selection event is violated. In first generation phage display applications where peptide sequencing was determined on cloned and isolated phages only (26,27), testing the randomness of a given library was typically superficial at best.

Recently however, next generation sequencing (NGS) (i.e. high-throughput parallel DNA sequencing) for the analysis of phage display peptides (23,25,28–66) has led to ‘next generation phage display’ (44,67). The massive increase in the number of peptides sequenced in a given experiment allowed for the first time to critically assess the quality, the randomness and deviations thereof of the libraries being used. In our previous study, we were surprised to discover that the presumably ‘random’ libraries employed are intrinsically ridden with severe deviations from randomness (23). In this initial study we adapted our ‘type 88’ fth1 vector (68) for NGS by incorporating the barcode and the nucleotide Adaptor-sequences upstream and downstream to the site corresponding to the peptide being displayed (thus producing the fth1-DP vector). This consequently led to the expression of the random peptides flanked by an additional 27 amino acids (the amino acid equivalents corresponding to the two Adaptor sequences and the barcode). NGS analysis of the peptide library revealed that in contrast to the expected, some phages in the library were dramatically over-represented (tens of thousands of copies). Closer examination of these deviant phages revealed that their inserts in the recombinant Protein 8 contained unintended stop codons that led to abortive termination and as a result a wild-type, as opposed to a recombinant phage phenotype. The fact that wild-type fth1 phages were generated despite having an abortively terminated recombinant Protein 8 is a property of the ‘type 88’ phage display systems where the genes encoding for both the wild-type and the recombinant Protein 8 are located on the same vector. Therefore, if one is missing, the phage would be assembled using the alternative Protein 8. This led to the hypothesis that a burden on phage assembly was imposed by the recombinant Protein 8 which was directly related to the length of the peptide being expressed (23,69,70). Hence, contrary to our belief, standard phage display libraries were unexpectedly distorted and far from the desired random and balanced peptide representation intended. Nonetheless, these less than optimal libraries were proven useful in laying the ground for NGS/phage studies as was published (23).

Other deviations might be the result of biased over-production or depletion of phages expressing specific peptides. This could be due to peptide conformations that could be particularly efficient for phage replication or alternatively impair phage assembly. Such biases have been proposed as ‘parasitic phages’ (29)—a phenomenon however that does not seem to be present in our libraries (23) (see below).

Here, we address the characterization of our phage display libraries themselves, focusing specifically on identifying what impacts the randomness of the peptides being displayed and provide practical correctives to produce markedly improved libraries for next generation phage display applications.

## MATERIALS AND METHODS

### Library construction

Libraries were constructed as previously described (26) and shown in Supplementary Figure S1. For this, two 5′ biotinylated oligonucleotides were used. The first contained the redundant ‘library’ sequence, (e.g. 6 × NNK - where N is any nucleotide and K is G or T) flanked by *Bgl1* sites compatible with the two *Sfi1* sites of the vector (e.g. 58 bases for 6 × NNK library). The second oligonucleotide, 18 bases, complemented the 3′ end of the first and was extended to ‘fill-in’ the complementary strand using Klenow polymerase. The product was digested with *Bgl1*, the short biotinylated segments were removed with streptavidin conjugated magnetic beads and the eluent was cloned into *Sfi1* digested fth1-DP or fth1 vector. This ligation mix was used to electroporate *Escherichia coli* as indicated in the ‘Results’ section followed by large scale phage production.

### Phosphoramidites calibration

The desired phosphoramidites ratio in the oligonucleotides used for the construction of the final library (fourth generation) were calibrated by The Midland Certified Reagent Company, Inc. as described in the text.

### Phage library production

The electroporated bacteria were grown in 100 ml TYx2/Tetracycline (20 μg/ml) medium overnight and then centrifuged at 8000 rpm, 20 min. The supernatants were transferred to 40 ml polyethylene glycol (PEG)/NaCl solution (33% PEG, 3.3M NaCl), incubated at 4°C overnight and centrifuged (8000 rpm, 45 min). The precipitated phages were re-suspended in a total volume of 10 ml Tris-buffered saline (TBS). The phages were precipitated again using 4 ml of PEG/NaCl (2 h, 4°C ice), centrifuged (8000 rpm, 45 min) and the phage pellets were re-suspended in 2 ml TBS. The re-suspended phages were centrifuged at 14 000 rpm for 10 min and supernatants were filtered through 0.45 μm microporous membrane filters.

### Sample preparation for Illumina sequencing

Two sets of experiments were performed. The pilot experiments used the fth1-DP library in which the Illumina adapters were cloned (libraries of first and second generations) as seen in the left panel of Supplementary Figure S2. Thus, the polymerase chain reaction (PCR) primers, corresponding to the Illumina adapters, were annealed directly to the phage DNA. A second set of optimized experiments utilized the wild-type fth1 libraries, not containing the Illumina adapters (libraries of third and fourth generations). In these cases, the adapters were added during the PCR as 5′ overhangs that corresponded to upstream and downstream phage DNA flanking the inserts as illustrated in the right panel of Supplementary Figure S2.

### Pilot experiments (libraries of first and second generations)

Parallel PCR reactions (50 μl) were prepared, each containing 10^10^ ϕ/ml phage library template (1 μl) in TBS, polymerase (*Taq*, Larova GmbH, VAR-04) and primers (primers one and two in Table 1) corresponding to the Illumina Adaptors A (i.e. Illumina Adaptor P5 and sequencing primer 1) and B (i.e. Illumina Adaptor P7 and sequencing primer 2). These Adaptors were previously inserted to create the fth1-DP vector. Each sample was amplified individually by a PCR reaction with the following thermal profile:

1. 95°C 5 min
2. 95°C 1 min
3. 53°C 1 min
4. 72°C 20 s
5. go back to step 2 × 34
6. 72°C 5 min

**Table 1.** Oligonucleotides used for Illumina sequencing

#	Name	Sequence (5′→ 3′)	Use
1	fth1-DP_F	AATGATACGGCGACCACCGAGATCTACACTCTTTCCCTACACGACGCTCT	PCR for sample preparation for Illumina
2	fth1-DP_R	CAAGCAGAAGACGGCATACGAGCTCTTCCGATCT	sequencing—fth1-DP libraries
3	fth1_Fp8_BC	AATGATACGGCGACCACCGAGATCTACACTCTTTCCCTACACGACGCTCTTCCGATCTNNNNNCAACGTGGC	PCR for sample preparation for Illumina
4	fth1_Rp8	CAAGCAGAAGACGGCATACGAGCTCTTCCGATCTGGCCCCAGAGGC	sequencing—fth1 libraries

The amplified PCR products were validated for size by running in 2% agarose gels. PCR samples were purified by RBC Real Genomics HiYield TM Gel/PCR DNA Fragments kit (RBCBioscience, YDF100) into 40 μl volume. The PCR cleaned products were pooled for every experiment and their concentration was measured. The pooled sample was dried by speed vac and sent for Illumina sequencing.

### Optimized experiments (libraries of third and fourth generations)

The PCR reactions were performed in the same manner as described for the pilot experiments but using different primers. The barcode and Illumina Adaptors A and B were added to the library as 5′ overhangs using the new primers (#3 and 4 in Table 1, Red colored Nts indicate the location of the 5mer barcode). The amplified PCR products were validated for size by running in 2% agarose gels. PCR samples were purified by Agencourt AMPure XP—PCR Purification (Beckman Coulter, A63881). The concentration of the PCR cleaned products was measured using a Qubit 2.0 fluorometer (Life Technologies, Q32866) diluted to 2nM and sent for Illumina sequencing.

### Illumina sequencing

Illumina NGS was performed at the National Center for Genome Resources (NCGR), Santa Fe, USA or at the Technion Genome Center (Haifa, Israel).

### National Center for Genome Resources, Santa Fe, New Mexico, USA

The NCGR kindly agreed to ‘lace’ their PhiX control lane with a small volume of our samples and helped us with the computational analyses. The dried samples were resuspended in 20 μl of elution buffer and analyzed using an Agilent BioAnalyzer 2100 to verify their quantity and quality. Quantitation for sequencing was done using qPCR, after which samples were normalized and 5 to 15 μl were spiked into 1000 μl (1.5 pM) of the Illumina PhiX control sample. Following clustering PCR, the sequencing was performed using an Illumina Genome Analyzer IIx.

### Technion Genome Center, Haifa, Israel

All sequencing with the fourth generation of random phage libraries was performed at the Technion Genome Center. A total of 11 pM of samples were used for clustering PCR followed by sequencing using HiSeq V4. The lane was spiked with 2% PhiX internal control for real time quality metrics during the sequencing.

### Pre-processing of sequence data

For all sequences 100% fidelity for a barcode reference sequence residing between the Adaptor A and first *Sfi1* site was mandatory (in sequence and reading frame). The sequences were further filtered out using the following criteria: (i) A sequence that contained one or more mismatches in the non-insert region was disqualified. (ii) The sequences with library inserts that deviated from the NNK codon were removed as this indicated frame shifted and aberrant phages. (iii) The library insert length was measured and sequences with non-expected lengths were removed.

### Phage growth in MC1061 versus DH5αF− bacteria

The experiment outline is described in Supplementary Figure S3. A fourth generation C10C library was cloned into the fth1 phage display vector as stated above and 10 μl of the ligation product was used to transform (heat shock) either MC1061 or DH5αF− bacteria (this was done in two independent repeats). The transformed bacteria were grown in 100 ml TYx2/Tetracycline (20 μg/ml) and the supernatants were collected after 1 and 19 h of growth in triplicates. From each supernatant a phage library was prepared as described in *Phage library production*. To prepare the samples for Illumina sequencing, 45 μl (concentrated to 1 μl) were collected from the libraries and used as templates for PCR. The libraries were then prepared for sequencing as descried in *Sample preparation for Illumina sequencing* - *Optimized experiments (libraries of third and fourth generations)*.

### Analyses of commercial Ph.D. library

The amino acid and nucleotide distributions of the naïve Ph.D.7 NEB library (lot number # 0061101) were based on a total of 3,960,150 sequences. This dataset was published by Derda *et al.* (29) as a supplementary file and was downloaded from: http://www.chem.ualberta.ca/∼derda/parasitepaper/rawfiles/NoMuPhD7-GTA-30FuR.txt.

## RESULTS

**Figure 1.**
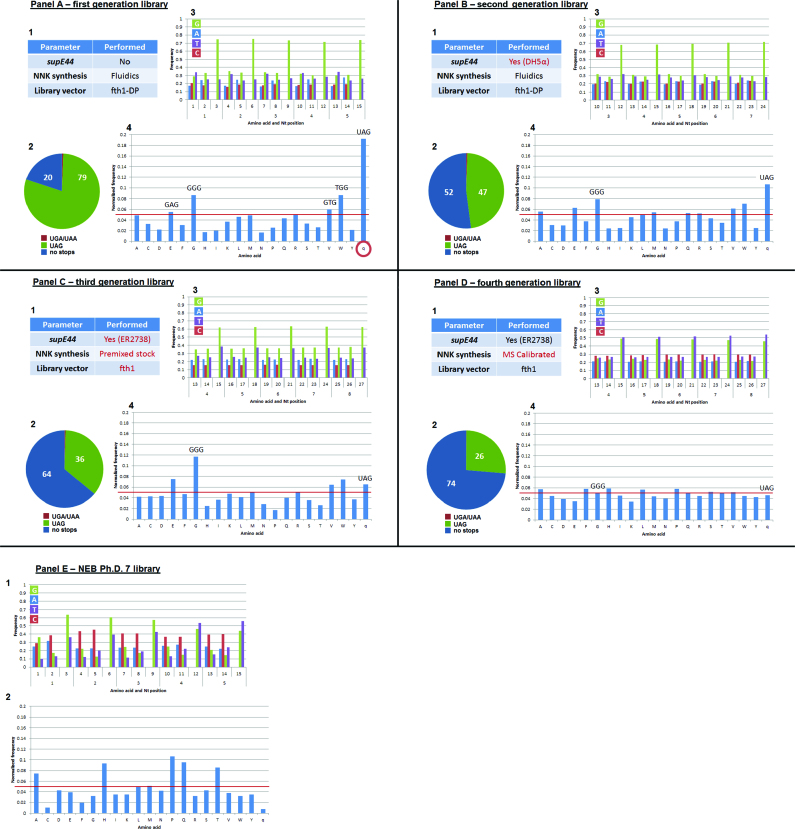
Distribution analysis of phage display peptide libraries. Oligonucleotides were synthesized and inserted into the fth1-DP (**A** and **B**) or fth1 (**C** and **D**) phage display systems and sequenced using Illumina NGS. (**1**) Parameters relevant to library construction. (**2**) Pie chart depicting the proportion of in-frame peptides in phage display libraries. The percentage of phages containing at least one of the UGA or UAA stop codons in their recombinant Protein 8 sequence is shown in red. Phages completely devoid of stop codons in their recombinant Protein 8 are shown in blue and peptides containing at least one UAG codon translated as glutamine are colored green. (**3**) The nucleotides distribution for five representative NNK codons. The oligonucleotides were constructed in an NNK format, where N position should contain equal 25% for each nucleotide and K position 50% of G and T. (**4**) The normalized amino acid distribution. The red horizontal line denotes the expected value (1/21 = 0.048, expected for 20 amino acids plus the additional glutamine which results from UAG suppression). (**E**) These data were collected from the commercially available Ph.D. 7 NEB library as described in text. (**1**) The nucleotide distribution for five representative NNK codons. (**2**) The normalized amino acid distribution.

The classic application of ‘phage display’ focused on isolating relatively few specific selected clones having the highest affinity for a given bait. So long as the affinities are sufficient and the general complexities of the libraries reasonable, there are good chances of isolating a lead phage for further development. However, ‘phage display’ has evolved to become a rich source of ‘big-data’, which consequently has afforded the opportunity to develop computational predictive algorithms. One of the assumptions made by such algorithms is that the ‘phage display’ libraries used for panning are genuinely random. To test if this assumption is valid we have further analyzed our previously published random libraries created by using the fth1-DP vector system (Figure 1A and B). In this way we have been able to measure the extent of the deviations from desired randomness of the peptides being displayed, while establishing a detailed base-line for comparisons and assessment of the efficacy of the proposed correctives described below.

**Figure 2.**
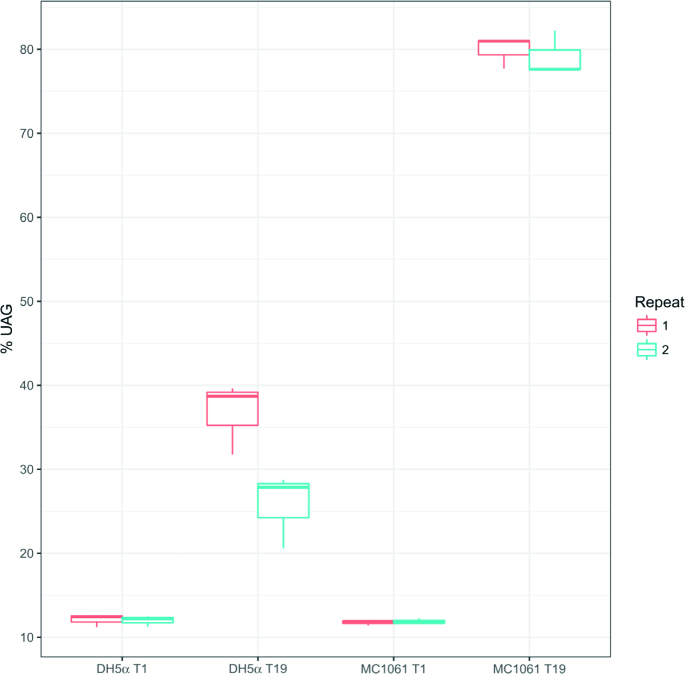
Comparison of UAG bearing phages in *supE44-* versus *supE44+* bacteria. A C10C phage library was grown in *supE44+* (DH5α) and *supE44−* (MC1061) in two independent repeats (red and cyan). Each library was sequenced three times after 1 and 19 h and the percentage of peptides carrying at least a single UAG codon is shown. Each boxplot represents three libraries taken from the same bacterial strain at the same time.

**Figure 3.**
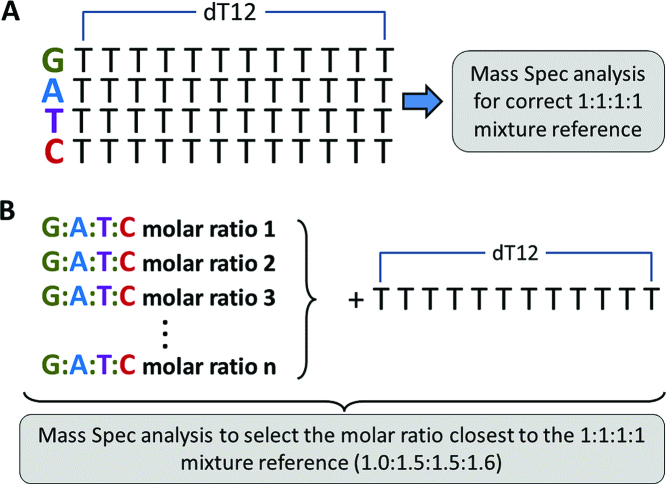
Oligonucleotide library calibration pipeline. (**A**) Baseline calibration. Four oligonucleotides comprised of 12 consecutive thymidines and a single additional oligonucleotide were prepared. Each oligo was HPLC purified, and mixed in the desired 1:1:1:1 molar ratio (for random N position in future library). The mixture was analyzed using mass spectrometry to be used as a correct molar ratio reference point, post synthesis. (**B**) Discovering the stock ACGT molar ratio that yields the desired equimolar post synthesis ratio. Thymidine 12mers were subjected to a single synthesis cycle with various ACGT ratio stock vials. To find the stock vial ratio giving equimolar synthesized ratio, the resulting oligonucleotides were Mass Spec analyzed to locate the one with the same profile discovered in (A), indicating a synthesized 1:1:1:1 molar ratio. The ratio found in (B) was used for future library production.

A first generation random 7 mer peptide library (Table 2) was constructed in the fth1-DP vector which was used to transform MC1061 *E. coli* strain for the first 24 hours (this strain is devoid of the *supE44* gene that suppresses the UAG stop codon (‘amber’ codon) by incorporating glutamine instead). The library was then amplified and maintained in DH5α cells that contain the *supE44* suppression gene translating UAG as glutamine and thus circumventing abortive termination and ensuring the production of functional recombinant Protein 8. The NNK codons were generated using the oligonucleotide-synthesizer's fluidics system (Figure 1A-1). The pie-chart in Figure 1A-2 illustrates the relative amounts of library phages with or without stop codons in their recombinant Protein 8. Figure 1A-3 shows a histogram illustrating the usage of the four nucleotides bases at each position of five representative codons of the random peptide insert (the full histogram is shown in Supplementary Figure S4A). In positions 1 and 2 of each codon all four nucleotides are present, while in position 3 only G and T exist, as to be expected when NNK is used for random peptide construction. However, surprisingly the stoichiometry was far from that intended. One would expect equimolar representation of G:A:T:C for N positions and G:T for K. G nucleotide is however, dramatically over represented while A and C are under-represented. This introduces an additional deviation from randomness and directly affects the amino acid compositions of the random peptides to be displayed. As is illustrated in Figure 1A-4, the surplus of G results in excess incorporation of amino acids E, G, V and W concomitant with a dramatic excess of glutamine due to UAG suppression, while other residues are consequently under expressed. We want to stress that the UAG bearing phages sequenced from this library gained their relative advantage over other non-UAG recombinant phages during the first 24 hours of library generation in *supE44-* bacteria, as they were assembled as wild-type fth1. However, the library was further maintained in DH5α bacteria which has the *supE44+* gene thus turning these phages back to glutamine bearing recombinant phages.

**Table 2.** Libraries characteristics

Library	Type	Theoretical complexity	Total number of peptides	Number of unique peptides
First generation	7	1.28E+09	116,516	112,153
Second generation	C8C	2.56E+10	57,871	57,845
Third generation	C10C	1.02E+13	2,475,659	1,898,920
Fourth generation	C10C	1.02E+13	2,048,929	1,484,655
NEB Ph.D.-7	7	1.28E+09	3,960,150	3,137,986

Note: The library titers for all four generations are similar. The differences in the ‘total number of peptides’ are the result of different number of phages sequenced for each library.

The construction of a second generation 8 mer library (Table 2) (23) by electroporation and 24 hours growth in DH5α cells that contain the *supE44* gene (instead of MC1061), reduced the number of peptides that contained the UAG amber codon (however, still not completely to the expected amount, ca. 27%). However, the effect of the biased incorporation of G and the consequent over representation of the corresponding amino acids were still evident (Figure 1B and Supplementary Figure S4B).

In light of these analyses, it is clear that there are a number of factors that impact on the randomness of the peptides displayed. The results thus far (Figure 1A and B) suggest that abortive-termination of the recombinant Protein 8 generates phages with a wild-type phenotype which gain a replicative advantage. To validate this assumption, we have experimentally followed the percentage of peptides containing UAG during phage library generation in bacteria with or without UAG suppression, i.e. DH5α and MC1061, respectively (Supplementary Figure S3). The ligated fth1-p8-C10C vector was transformed into MC1061 or DH5α bacteria and the UAG fraction was measured after 1 and 19 h of growth. While the percentage of UAG was similar after 1 h (median of 12.04 and 11.82% for the DH5α and MC1061 strains, respectively) a substantial difference of over 40% was observed after 19 h (Figure 2). These results demonstrate that production of libraries in bacteria that are devoid of suppression of amber stop codon (i.e. *supE44*) leads therefore to over representation of phages containing UAG bearing inserts.

As is illustrated in Figure 1A-2 and B-2, production of libraries in *E. coli* strains containing *supE44* reduces the effect of UAG to some degree (79 to 47% UAG containing phages). However, the overabundance of UAG containing phages is indicative of less than 100% efficiency of UAG suppression by *supE44* (71) as also demonstrated by the rise of the frequency of peptides containing UAG after 19 h of growth in DH5α bacteria (Figure 2). Hence, further steps need to be taken to reduce the assembly burden imposed by the recombinant Protein 8. One way of accomplishing this could be by reducing the length of the insert being expressed by the recombinant Protein 8 in the fth1-DP system. For that, elimination of the 27 amino acids corresponding to the barcode and the 5′ and 3′ Illumina Adaptor sequences was proposed. This, in theory, should reduce the relative advantage of phages bearing the UAG stop codon as it has been shown that the burden imposed upon the phage correlates with the length of the recombinant protein being expressed (69,70).

The second factor that impairs randomness is a marked imbalance in incorporation of the G nucleotide in comparison to the others. This is particularly relevant in the K position causing an over-representation of amino acids with G in their third codon position.

In order to rectify these problems, we tested three modifications intended as correctives for improved third generation library construction:

- Initially, the NNK mixtures were made automatically through a mixing regimen programed by the oligonucleotide-synthesizer generating presumably equimolar mixtures of phosphoramidites taken from four individual stock solutions of G, A, T and C. As a corrective intended to ensure that the N and K mixtures were truly consistent and precise, two equimolar stock solutions of hand-mixed GATC and GT were prepared and used in synthesizing the NNK segments of the oligonucleotides produced for library construction.
- The electroporation was performed using the K12 ER2738 strain of *E. coli* (NEB, E4104S) which is a high efficiency electroporation strain that contains the *supE44* gene.
- The libraries were constructed in the standard fth1 vector so as to reduce the amino acid residue burden of the recombinant Protein 8 displaying the random peptides (Supplementary Figure S2). Consequently, the A and B Adaptor sequences were introduced via the PCR amplification step of sample preparation using primers that corresponded to the fth1 sequences preceding and following the *Sfi1* sites and containing the Adaptor sequences as 5′-end extensions (see ‘Materials and Methods’ section).

In the third generation library (Table 2) the percentage of unique sequences devoid of stop codons increased from 52 to 64% and correspondingly the UAG containing sequences dropped from 47 to 36% (Figure 1C-2). The distorted GT usage improved, however only slightly, and was still prominent. These improvements did generate a more balanced amino acid composition of peptides yet the residues that contained G in their third position continued to be over expressed. Note also that removal of the excess 27 amino acids corresponding to the 5′ and 3′ Adaptors seems to reduce the effect of the assembly burden imposed by recombinant Protein 8 and as a result the relative amount of UAG containing inserts is only slightly elevated above the expected value (ca. 27%). Therefore, although the adapters were included in the original fth1-DP vector with the thought that it would be useful, this experiment illustrates that although this vector works, it poses some problems and has no advantage and thus has been abandoned.

Although these measures did improve the profile of peptides in the library, the excess incorporation of the G phosphoramidite was still apparent and not resolved by the manually prepared stock equimolar mixtures used. Hence, we postulated that the distortions may be caused by variations in efficiency of chemical incorporation for each phosphoramidite. In order to address such variance in chemistries we critically calibrated the phosphoramidite mixtures with an intent to compensate for differences in incorporation efficiencies.

For this the following steps were taken:

- Oligonucleotides containing 12 consecutive thymidine residues (T12) were prepared. Then a single residue was added and four separate 13 mer oligonucleotides (G-T12, A-T12, C-T12 and T-T12, Figure 3A) were purified by HPLC and analyzed by mass spectroscopy to generate a defining spectrum for each construct.
- Equimolar mixtures using the four 13 mer oligonucleotides were carefully prepared and measured by mass spectroscopy. This provided a calibrated base line spectrum that represented a true 1:1:1:1 mixture for G:A:T:C and a 1:1 mixture of G:T.
- A panel of variable molar mixtures of G, A, T and C phosphoramidites and G, T phosphoramidites were prepared and used as a source stock solution to incorporate single residues to the T12 oligonucleotide (Figure 3B).
- The resulting 13 mer oligonucleotide products for each mixture tested were HPLC purified and analyzed by Mass Spectroscopy and the profiles were compared to the calibrated base line spectra generated in Step (ii).
- The mixture prepared in Step (iii) that gave the closest result most similar to the calibrated base line of equimolarity was then used to prepare oligonucleotides for subsequent library construction. The molar ratios that produced the best equimolar distribution of G, A, T and C in the oligonucleotides were: N = G:1.0, A:1.5, T:1.5 C:1.6 and K = G:1.0, T:1.5.
- Fourth Generation libraries were constructed using the HPLC purified and calibrated oligonucleotides of Step (v) and the fth1 vector and ER2738 *E. coli* strain for electroporation and then analyzed by NGS (Table 2).

Figure 1D demonstrates that these last measures taken to improve library construction and true unbiased amino acid representation had a dramatic positive effect. Not only did the amino acids previously over represented, drop to a reasonable expected base line, but the correction for G overuse reduced the UAG concentration as well. To assess the corrections taken to improve the randomness of our libraries we analysed the profile produced for the commercially available New England BioLabs (NEB) Ph.D.-7 library (see, for example (2,29,47,49,53,57,63,65,66,72–75)). For this, we have compared the nucleotide and amino-acid distributions of the NEB library with that observed in our fourth generation unbiased library (Figure 1D). The results demonstrate that the commercially available library is substantially deviated from randomness both at the nucleotides (Figure 1E-1 and Supplementary Figure S4E) and the amino acid (Figure 1E-2) levels. Specifically, at the nucleotide level, G reached over 60% in some of the K positions, while the amino acids ranged between 1 and 10% frequencies. Interestingly, the biases in the Ph.D. 7 library differ from the biases observed in our first to third generation libraries. This difference could be explained by the fact that these libraries are constructed in a different system. While the Ph.D 7 library is constructed in the pIII minor coat protein of M13 phage, our libraries were built in the PVIII major coat protein of fth1, which is a derivative of the fd phage (68). Nevertheless, we do believe that the biases in all phage display systems could be fixed by adopting the methodology suggested above.

## DISCUSSION

Over the years, a variety of algorithms designed to predict epitope structures have been published (see review by Sun *et al.* (22)). Common to all these procedures is the use of affinity selected peptides as input data. The presumption in all these applications is that the selected peptides are derived from biopanning of *bone fide* unbiased random peptide libraries. This same presumption is central to the use of phage display peptide libraries in the analysis of polyclonal sera. The intrinsic need to evaluate hundreds and thousands of peptides to best study the multiplicity of antibodies in polyclonal serum has stimulated the implementation of NGS to phage display analyses. This has for the first time allowed us to also critically examine the degree of randomness of the peptides displayed at the onset of a given procedure. To our surprise we found that, despite our intention, the libraries being used were far from random. In our initial publication we noted the over-representation of phages containing the UAG stop codon in their recombinant Protein 8, and proposed using *supE44* containing *E. coli* strains from the very first steps of electroporation in library construction (23). Here, we validate this assumption by showing that the UAG fraction increases extensively over time in libraries generated in *supE44-* bacteria. This phenomenon is not observed in libraries created in *supE44+* bacteria. Moreover, we further examine the quality of random peptide libraries, discover distortions and provide remedies.

The burden of incorporating recombinant Protein 8 displaying N terminal fusion peptides during the course of assembly is apparent. Hence, we decided to lessen the burden and remove the Adaptor sequences that flanked the cloning cassette in the fth1-DP vector originally proposed. The introduction of the Adaptors via the PCR step in sample preparation proved efficient and so NGS could be performed using any of our former libraries constructed in the fth1 vector. Indeed PCR incorporation of Adaptor sequences is routine for most phage-NGS protocols (25,28,76).

However, measuring the relative concentration of the four nucleotide bases at each position of the NNK codons revealed that there are serious distortions in the efficiency of phosphoramidite incorporation during the course of oligonucleotide synthesis. Thus, incorporation of G appears to be markedly more efficient as compared to A, C or T. The ramification of this is that the amino acid compositions are strongly biased for those residues containing G. Such biases in the chemistries have previously been reported in generation of aptamer libraries (77,78). To alleviate such biases it was suggested for example to use manually mixed stocks via a single port delivery system although this approach did not fully resolve the bias (29). No clear chemical basis explaining why G is preferentially incorporated in our libraries could be found in the nucleic acid chemistry literature. Therefore, we took an empirical approach. The detailed and precise calibration using Mass Spectroscopy in our study proves effective. After determining the spectrum that represents carefully stoichiometrically balanced mixtures, we constructed a library using different mixtures of nucleotides until a comparable spectrum was obtained. Although we cannot explain the chemical mechanisms underlying the biased incorporation of G in each position, our practical approach provided an effective solution in overcoming this distortion.

The calibrated nucleotide-ratios were used to construct eight additional random phage display calibrated libraries. The libraries were of four different lengths (6mer, 8mer, 10mer and 12mer), either linear or cysteine looped to allow constrained looped peptides as well as linear ones: 6, 8, 10, 12, C6C, C8C, C10C, C12C. All eight libraries were pooled to generate a single library designated the ‘mixed adjusted-complexity library (6, 8, 10, 12, C6C, C8C, C10C, C12C)’ (Supplementary Table S1). The mixing was adjusted so that every library in the mixture had the same complexity *(i.e.*, each unique peptide had the same number of copies in the mixed library, regardless of its length) (Supplementary Table S1). This library is used routinely in our on-going research.

The non-biased peptide libraries generated in this study set a new standard for randomness in combinatorial phage display libraries compared to previously published libraries including the commercially available Ph.D. libraries (NEB). This is especially important when analyzing ‘big data’ of peptides obtained using next generation phage display and when using peptides for the computational prediction of target epitopes.

## DATA AVAILABILITY

The sequence datasets of the libraries described above (both raw reads and translated peptides) are available as supplementary information at Dryad: https://doi.org/10.5061/dryad.8ks16

## Supplementary Material

Supplementary Data
