# Supplementary material for: Phage display peptide libraries: deviations from randomness and correctives
Source: Nucleic Acids Res. 2018 Feb 6;46(9):e52. doi: 10.1093/nar/gky077 (PMC5961013; doi:10.1093/nar/gky077)
Supplement: Supplementary Data [file gky077_supp.docx]

# Supplementary information for:

# Phage Display Peptide Libraries: deviations from randomness and correctives

Supplementary tables

Supplementary Table S1: Fourth generation libraries characteristics

| **Library** | **Type** | **Theoretical complexity** | **Measured complexity** | **Average copy number in top 5% of pooled library** |
| --- | --- | --- | --- | --- |
| **4^st^ generation** | 6 | 6.40E+07 | 2.40E+09 | 5.870 |
| **4^st^ generation** | C6C | 6.40E+07 | 2.00E+10 | 5.562 |
| **4^st^ generation** | 8 | 2.56E+10 | 1.60E+09 | 5.914 |
| **4^st^ generation** | C8C | 2.56E+10 | 6.00E+09 | 5.714 |
| **4^st^ generation** | 10 | 1.02E+13 | 6.00E+09 | 5.789 |
| **4^st^ generation** | C10C | 1.02E+13 | 5.00E+09 | 5.918 |
| **4^st^ generation** | 12 | 4.10E+15 | 5.60E+09 | 5.841 |
| **4^st^ generation** | C12C | 4.10E+15 | 5.60E+09 | 5.870 |

Supplementary Table S1. Fourth generation libraries characteristics

The characteristics of the eight libraries comprising the final pooled library are given. The libraries are of four different lengths with or without flanking cysteines. The theoretical complexity was calculated as the maximal number of possible different peptide sequences of that length. To construct these libraries we have cloned the eight fourth generation NNK libraries into the fth1 vector. The cloned vectors were electroporated into ER2738 *(supE44+)* bacteria and the total number of transformants was measured (by plating a dilution of the electroporated library) after one hour of growth to represent the maximum complexity of each library (“Measured complexity”). All eight libraries were pooled into a single library taking into account their relative theoretical and measured complexities to generate a single library such that every peptide will be present in the same number of copies. This library, “mixed adjusted-complexity library (6, 8, 10, 12, C6C, C8C, C10C, C12C)”, was sequenced 6 times. The average copy number of each library type in the 5% most frequent peptides of the pooled library is reported.

**Supplementary Figures**

***Bgl1***

***Bgl1***

***6×(NNK)***

58 bp

18 bp

5’

5’

**Supplementary Figure S1. Library construction.** A scheme describing the preparation of a 6mer library (orange) before ligation into the fth1 or fth1-DP vector. Two 5’ biotin (red dot) labeled oligonucleotides (black lines) were annealed using an 18 overlap region (vertical lines). After completion with Klenow polymerase (dashed – 58 bp product) the library was digested with *Bgl1* restriction enzymes (blue) to give compatible ends for fth1 or fth1-DP digested with *Sfi1*.

**
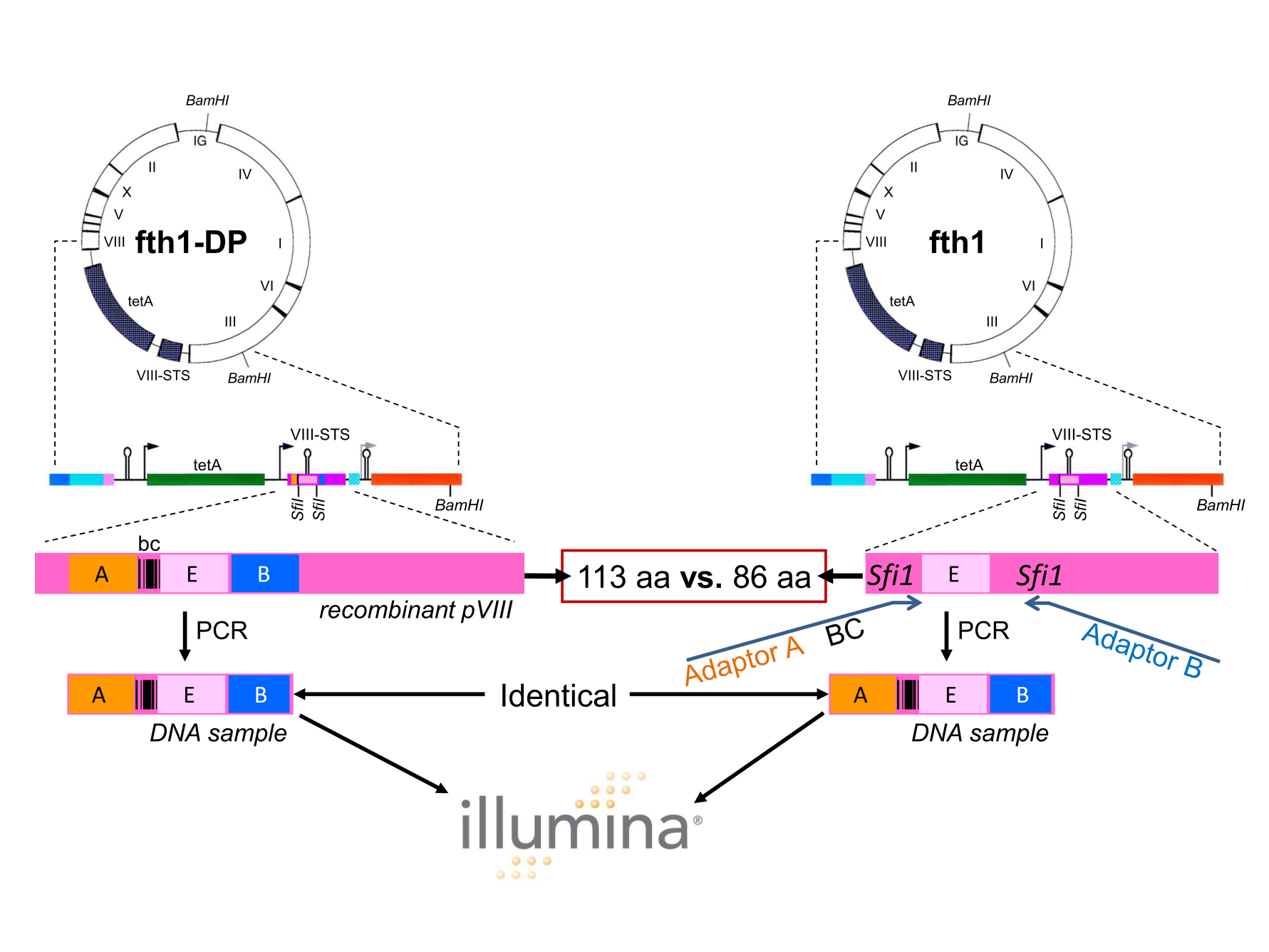
Supplementary Figure S2. Schemes of recombinant protein 8 in fth1-DP vs fth1.** fth1-DP differs from fth1 in its recombinant pVIII gene (*VIII-STS*). Adaptors A (orange) and B (blue) were inserted to the recombinant pVIII fragment of fth1 to create fth1-DP, resulting in a 113 amino acid protein compared to 86 aa in fth1 for a 6mer insert. The PCR step in fth1-DP utilizes primers that anneal to the Illumina adaptors while in fth1 the primers are annealed to the *Sfi1* sites, adding the adaptors by their 5’ non annealed end. The PCR products in both cases are identical and are ready for Illumina NGS. BC denotes the samples barcode allowing multiplex sequencing.

**
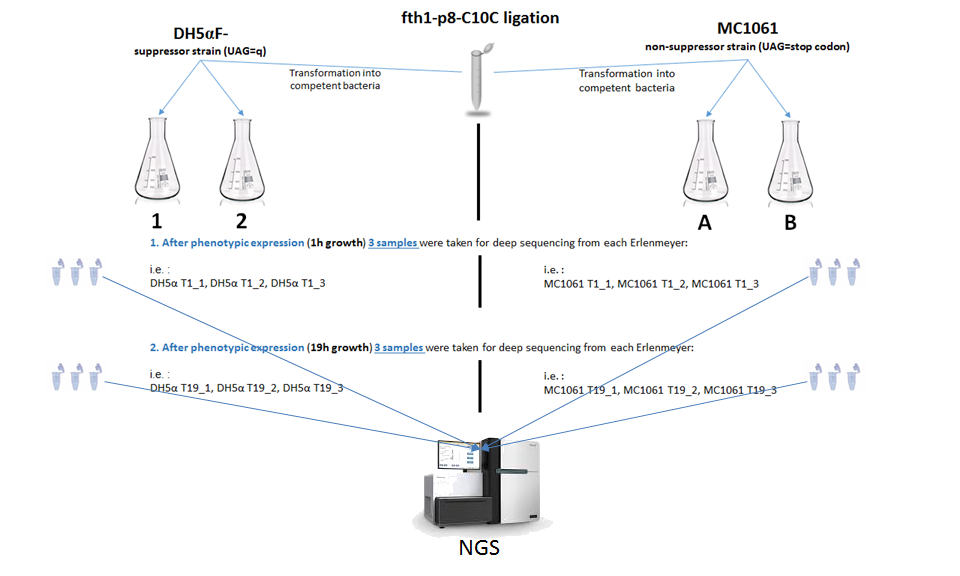
**

**Supplementary Figure S3. A scheme of phage growth in MC1061 vs. DH5αF- experiment.** A 4^th^ generation C10C library was cloned into the fth1 phage display vector and the ligation product was used to transform (heat shock) either MC1061 or DH5αF- bacteria in two independent repeats (**1** and **2** for DH5αF- and **A** and **B** for MC1061). The transformed bacteria were grown and the supernatants were collected after 1 and 19 hours in triplicates. From each supernatant a phage library was prepared and sent to Illumina NGS sequencing to discover the frequency of UAG containing phages (see ‘MATERIAL AND METHODS’ for details).

**Supplementary Figure S4. Nucleotide distribution in each position of the random library**. (A) 1^st^, (B) 2^nd^, (C) 3^rd^, (D) 4^th^ generation and (E) NEB Ph.D-7 phage display random libraries (see main text for additional details about each library).**
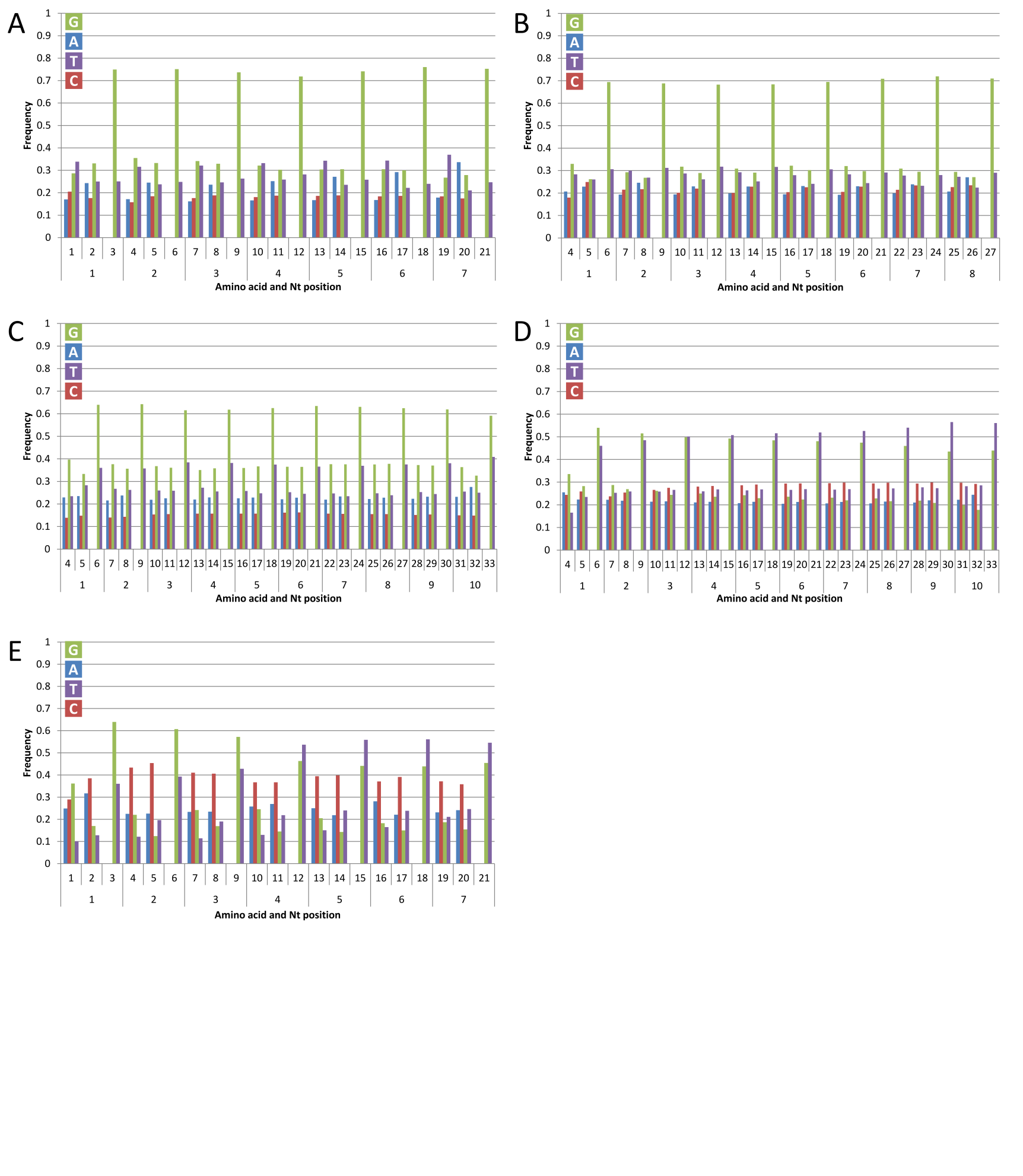
**
